# Advances in androgenetic alopecia model research

**DOI:** 10.3389/fphar.2026.1935819

**Published:** 2026-09-29

**Authors:** Mingrui Zhang, Guiyun Zhang, Wensi Zhang, Yihe Zhang, Yangchun Xu

**Affiliations:** 1 Department of Dermatology, The Second Hospital of Jilin University, Changchun, China; 2 School of Biological and Food Engineering, Jilin Engineering Normal University, Changchun, China

**Keywords:** androgenetic alopecia, *ex vivo* models, hair follicle, *in vitro* models, *in vivo* models, research progress

## Abstract

Androgenetic alopecia (AGA) is the most prevalent form of non-scarring hair loss, and its pathogenesis is quite complex, involving androgen metabolism, follicular microenvironment imbalance, oxidative stress, and inflammatory responses. Understanding these multifactorial mechanisms is critical for developing effective therapeutic strategies. To this end, various experimental models have been extensively employed. In this review, we summarized recent progress in AGA model research, covering *in vitro* cell models such as dermal papilla cells and keratinocytes, *in vivo* animal models—primarily mice treated with testosterone or dihydrotestosterone, as well as genetically modified strains, and *ex vivo* hair follicle organ culture models. Some emerging approaches, like three-dimensional organoid systems and Organ-on-a-Chip Technologies, are also discussed. The aim of this review is to provide a theoretical framework to guide future drug development and mechanistic studies in AGA.

## 1 Introduction

Androgenetic alopecia (AGA), often referred to as male or female pattern hair loss, is the most prevalent form of progressive, non-scarring hair loss worldwide, affecting a significant proportion of the adult population. Pathologically, it is characterized by the miniaturization of hair follicles, a shortened anagen phase, and a reduction in follicular density; these changes lead to patterned hair thinning, while varying between sexes. It imposes a considerable cosmetic and psychological burden ultimately (Devja et al., 2023). Despite its high prevalence, it must be said that the precise pathogenesis of AGA remains incompletely understood. The current consensus points to a rather complex interplay, involving genetic predisposition, well-known hormonal influences, and perhaps less understood local factors within the follicular microenvironment.

Perhaps one of the central aspects in the pathophysiology of AGA is the aberrant androgen metabolism within hair follicles, especially the involvement of dihydrotestosterone (DHT), a potent androgen derived from testosterone via the activity of 5-alpha reductase. DHT binds to androgen receptors (AR) in dermal papilla cells (DPCs), then triggers signaling cascades that ultimately lead to follicular miniaturization and disruption of the hair cycle (Fu et al., 2021). It is also worth noting that these AR-mediated effects are further modulated by genetic factors. Genome-wide studies have identified certain locis which are linked to AGA susceptibility, such as TSNARE1-involved in vesicle transport, FZD1-a key regulator of the Wnt signaling pathway, GJC1-a gap junction protein. And it is possible that these genetic differences may differentially influence male versus female pattern hair loss (Lee et al., 2024). Moreover, the role of androgens in female AGA is less clearly defined compared to in males. Some studies suggest that in affected women, the free androgen index might serve as a more sensitive biomarker for hyperandrogenism than measuring testosterone or sex hormone-binding globulin levels alone (Hosny et al., 2026).

Beyond androgenic regulation, the pathogenesis of AGA is also substantially influenced by the follicular microenvironment and systemic factors. For instance, oxidative stress, inflammation and a state of dysregulated autophagy within the perifollicular region, have been implicated in follicle degeneration and impaired regeneration (Yang et al., 2024). The accumulation of reactive oxygen species (ROS) leads to vascular dysfunction, which in turn compromises the viability of hair follicles. This emphasizes the importance of maintaining redox homeostasis for hair follicle health. Recent studies have shown that therapeutic strategies which target oxidative stress and simultaneously promote angiogenesis can help to rejuvenate the perifollicular environment, thereby creating conditions more favorable for hair regrowth (Yang et al., 2024). Additionally, research shows that the expression of hypoxia-inducible factor-1α within DPCs plays a role in regulating trichogenic gene expression, suggesting hypoxic signaling pathways are involved in regulating hair follicle cycling and may represent promising therapeutic targets (Seo et al., 2023).

The genetic and molecular complexity of AGA largely necessitates the use of robust experimental models. Traditional two-dimensional cell cultures of human dermal papilla cells (HDPCs) and C57BL/6 mouse models have been extensively utilized for many years. We rely on these models to dissect the underlying mechanisms and evaluate novel treatments. The primary role has been to study AR activation by DHT and to map its downstream effects on hair follicle regression and miniaturization (Fu et al., 2021). It is worth noting that these systems have successfully elucidated several key pathways, such as the AR-mediated delay in hair regrowth and morphological changes which are consistent with what is observed in clinical AGA. However, it’s also true that these models come with notable limitations, particularly in terms of their clinical relevance and reproducibility. These shortcomings have driven the development of more sophisticated systems, including three-dimensional organoid cultures and *ex vivo* hair follicle organ cultures, which are designed to better recapitulate the follicular microenvironment and hair cycle dynamics (Fu et al., 2021). Furthermore, animal models, mainly murine systems, have been employed to investigate hormonal influences and to test therapeutic interventions.

Current FDA-approved therapies for AGA are limited to topical minoxidil and oral finasteride. Although both are widely used, it is important to note that their efficacy is often modest, and associated with side effects, restrict long-term adherence. Finasteride, which is a 5-alpha reductase inhibitor, works by reducing DHT synthesis. However, it may also cause some systemic adverse effects, which has prompted considerable interest in developing topical formulations. Another drug worth mentioning here is dutasteride, a different 5-alpha reductase inhibitor with broader isoenzyme inhibition. Some studies have suggested that it may demonstrate superior efficacy compared to finasteride, although its safety profile still requires further evaluation (Almudimeegh et al., 2024). Given these limitations, emerging therapies, including platelet-rich plasma (PRP), low-dose oral minoxidil, and novel compounds targeting oxidative stress or inflammation, have shown promise, offering potential adjuncts or alternatives treatments.

It is true that significant progress has been made, but translating these experimental findings into effective clinical therapies still presents considerable challenges. The sex-specific differences in AGA pathogenesis, the multifactorial nature of follicular miniaturization, and the limitations of the models all call for continuous optimization of experimental systems and the development of personalized treatment strategies. Systematic reviews and meta-analyses highlight an urgent need for high-quality randomized controlled trials, especially involving female patients, who are often underrepresented in clinical research, to validate the safety and efficacy of emerging therapies. Furthermore, the integration of genomic profiling with molecular diagnostics potentially enables more personalized treatment strategies for patients.

To conclude, AGA is a multifactorial condition, and experimental models have been pivotal in uncovering its mechanisms and assessing potential treatments. This article provides a narrative review of the latest advances in AGA models, covering *in vitro* cell systems, *in vivo* animal models, and *ex vivo* hair follicle organ culture techniques (Figure 1). It also discusses emerging approaches, such as three-dimensional organoids and organ-on-a-chip technologies. We further evaluate the advantages and limitations of various models (Table 1). Additionally, the article offers several perspectives on future directions—with the aim of providing a comprehensive reference for selecting appropriate models in both basic and clinical AGA research.

**FIGURE 1:**
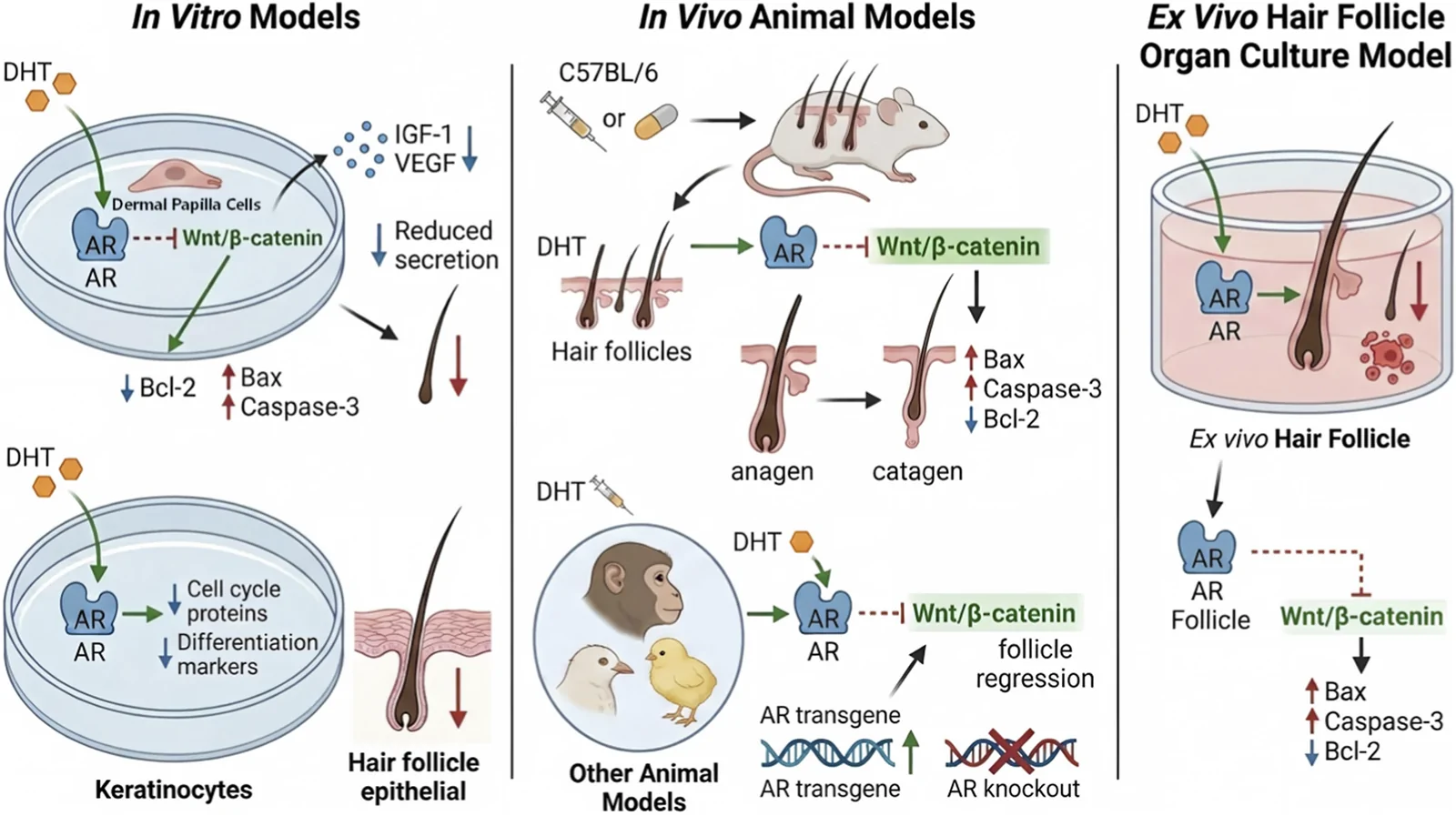
Experimental models and molecular mechanisms in AGA. This diagram depicts the three primary experimental models used in AGA research—*in vitro* models, *in vivo* animal models, and *ex vivo* hair follicle organ culture models. It emphasizes the antagonistic interaction between the androgen receptor (AR) and the Wnt/β-catenin signaling pathway, as well as the resulting apoptosis and degenerative changes in hair follicles.

**TABLE 1:** Systematic evaluation of AGA models based on the key dimension of similarity to human pathology.

Model type		Key features	Primary use	Advantages	Limitations
*In vitro* cell models		Highly simplified; focus on androgen receptor (AR) signaling	Dissecting molecular mechanisms; initial drug target screening	Easy operation, low cost, rapid large-scale drug screening, strong controllability, direct mechanistic study	Require validation in more complex settings
*In vivo* animal models	Mouse	Replicate core androgen-dependent processes; cost-effective	Primary choice for *in vivo* mechanistic research	Well-defined genetic background, rapid reproduction, low cost	Lower pathological similarity to humans
	Non-human Primate	Closest pathological phenotype and treatment response similarity to humans	High translational relevance	High physiological and genetic homology with humans, accurate drug evaluation	Practical constraints prevent widespread use
	Avian	Support broad hormonal regulation principles	Limited insight into AGA-specific pathology	Similarities in feather and hair follicle cycles	Offer limited pathological specificity
*Ex vivo* hair follicle organ culture		Uses intact human tissue; mirrors native hair follicle structure	Bridge between cellular and animal studies; high clinical predictive value	Preserves 3D structure, cell types, and ECM; recapitulates hair cycle; high human predictive value	Dependent on human tissue availability

## 2 *In vitro* cell models

### 2.1 Dermal papilla cell models

DPCs play a central regulatory role in hair follicle development and cyclic regeneration, and their dysfunction is a key factor in the pathogenesis of androgenetic alopecia (AGA). To study the direct impact of androgens on hair follicles, *in vitro* cultured HDPCs are extensively used, with DHT or testosterone applied to mimic the pathological conditions of AGA (Liu et al., 2024).

DHT exposure has been experimentally shown to induce senescence, apoptosis, and proliferation inhibition in HDPCs. This is accompanied by downregulation of Wnt/β-catenin signaling pathway proteins, which are essential for maintaining hair follicle size and function. Consequently, these alterations lead to follicular miniaturization, which is the most important characteristic feature of AGA (Park et al., 2025). For example, the antioxidant oxyresveratrol alleviates H_2_O_2_-induced oxidative stress in HDPCs by reducing ROS and pro-inflammatory cytokine production, which helps maintain cellular function (Tran et al., 2025). Moreover, HDPCs models have been instrumental in evaluating the therapeutic potential of exosomes, natural compounds, and gene therapy vectors. Notably, exosomes derived from adipose mesenchymal stem cells (ADMSC-Exos) exert protective effects against DHT-induced damage in HDPCs by delivering microRNAs such as miR-574–3p and miR-125a-5p, which target the TGF-β1/SMAD2 signaling axis, reversing cellular injury (Ma et al., 2025).

These findings underscore the utility of HDPCs model in elucidating androgen-mediated molecular mechanisms and screening novel interventions aimed at restoring DPCs function and hair follicle integrity in AGA.

### 2.2 Keratinocyte models

Keratinocytes of the hair follicle, particularly those constituting the outer root sheath, are the primary cellular components forming the hair shaft and are subject to androgenic regulation. In AGA, DHT induces apoptosis in follicular keratinocytes, accelerating the transition from the anagen phase to the catagen phase, thereby contributing to hair follicle miniaturization and hair loss. *In vitro* cultures of human hair follicle keratinocytes, including immortalized HaCaT cells and primary keratinocytes, are widely employed to dissect the mechanisms underlying androgen-induced cell death.

Studies reveal that activation of NADPH oxidase (Nox) isoenzymes plays a crucial role in mediating DHT-induced oxidative stress and apoptosis in keratinocytes (Begum et al., 2022). The natural compound 1′S-1′-acetoxy-oxyphenylpropyl acetate has been demonstrated to inhibit Nox activity, thereby reducing H_2_O_2_ generation and protecting keratinocytes from oxidative damage (Park et al., 2025). Additionally, keratinocyte models facilitate the evaluation of therapeutic agents modulating the follicular microenvironment. For example, PRP enhances autophagy in keratinocytes, promoting cell survival and potentially counteracting androgen-induced follicular regression (Xiao et al., 2025).

These models not only provide a platform for testing compounds that preserve keratinocyte viability and function in AGA, but also offer crucial insights into the cellular and molecular events triggered by androgens in keratinocytes (Figure 2).

**FIGURE 2:**
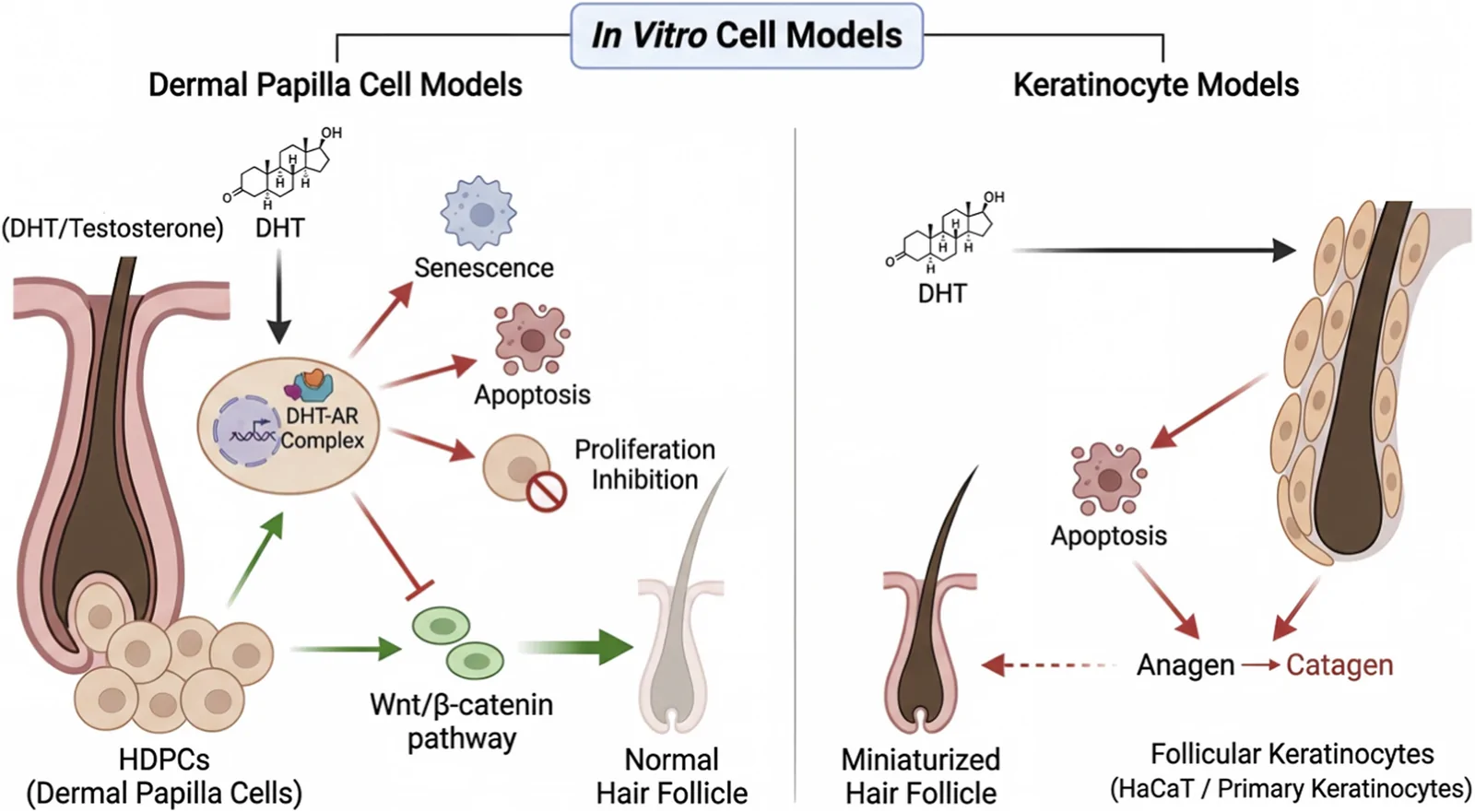
*In vitro* cell models of AGA and the pathogenic mechanisms. This diagram illustrates two major *in vitro* cell models used to study AGA—the dermal papilla cell model and the keratinocyte model. It highlights how DHT or testosterone, acting through AR-mediated intracellular signaling pathways, differentially targets dermal papilla cells and hair follicle epithelial cells, ultimately leading to hair follicle miniaturization and disruption of the hair cycle.

### 2.3 Advantages and limitations of *in vitro* models

*In vitro* cell culture models demonstrate significant advantages in AGA research. They are easy to operate, low-cost, and allow for rapid large-scale drug screening and toxicity assessment. Moreover, they offer strong controllability, enabling precise regulation of various parameters in the culture environment, such as DHT concentration, culture time, oxygen partial pressure, etc., making them direct tools for studying cell proliferation, apoptosis, cell cycle arrest, and specific signaling pathways (e.g., Wnt/β-catenin, TGF-β/SMAD). Additionally, two-dimensional models also support efficient molecular analysis using techniques such as Western blot and qRT-PCR, aiding mechanistic insight into AGA.

However, these models have clear limitations. A major drawback is the absence of three-dimensional cell–cell and cell–extracellular matrix interactions. *In vivo*, hair follicles are highly complex three-dimensional structures, where dermal papilla cells interact with keratinocytes, melanocytes, immune cells, and the ECM through paracrine and physical signaling—key for follicular cycling and function. *In vitro* cell models cannot simulate this microenvironment, leading to potential alterations in cell morphology, polarity, gene expression profiles, and signal transduction methods. Furthermore, two-dimensional models cannot reproduce the full hair cycle stages (anagen, catagen, telogen), whereas AGA involves cycle shortening and follicular miniaturization.

Therefore, although two-dimensional models are indispensable for mechanism exploration and initial screening, their results need to be interpreted with caution and validated in combination with more complex *in vitro* three-dimensional models or *in vivo* animal models to more accurately reflect the pathophysiological process of AGA (McElwee and Sundberg, 2025).

## 3 *In vivo* animal models

### 3.1 Mouse model

Mouse models are generally considered the most widely utilized animal systems for research into AGA. This widespread use stems from several practical advantages: rapid reproduction, low costs, well-defined genetic backgrounds, and ease of experimental manipulation.

A common approach to inducing an AGA-like phenotype in mice is through the administration of androgens (e.g., testosterone or DHT). These are usually delivered via subcutaneous or intraperitoneal injections. The intended effect is to mimic the androgenic environment associated with human AGA, as closely as possible. Because of their dorsal skin displays a well-characterized hair cycle and responds to androgen treatment in a relatively predictable manner, C57BL/6J mice are often regarded as a kind of standard for AGA modeling. In these models, the administration of androgens following depilation typically leads to a range of observable effects: delayed hair regrowth, follicular miniaturization, reduced hair length, and a decrease in follicle numbers. Collectively, these changes thereby recapitulate key pathological features characteristic of human AGA (Fu et al., 2021). Furthermore, genetically modified mouse models have been employed to dissect molecular mechanisms underlying AGA. For instance, TRPV3 knockout mice have been used to study the role of TRPV3 channels in hair growth regulation. The natural compound Forsythiaside A appears to selectively inhibit the TRPV3 channel. In these models, this action alleviates the hair growth suppression induced by DHT. This finding thereby highlights the importance of specific molecular targets in AGA pathogenesis (Wang L. et al., 2025). Besides, the research also involves gene-mutant mouse models and xenograft mouse models. The former utilizes animals with spontaneous hair loss or sparse hair phenotypes, with a typical representative being the balding mouse (B6. Cg-Tg(KRT14-AR)8Yz); this strain carries a human AR cDNA transgene driven by the human keratin 14 promoter, rendering hair follicles highly sensitive to androgens. The latter refers to the transplantation of heterologous tissues (such as human skin) into immunodeficient mice. Among these, the two spontaneously mutant strains, the nude mouse (Foxn1nu) and the hairless mouse (Hr), are often used in dermatological research due to their hair loss characteristics (Figure 3).

**FIGURE 3:**
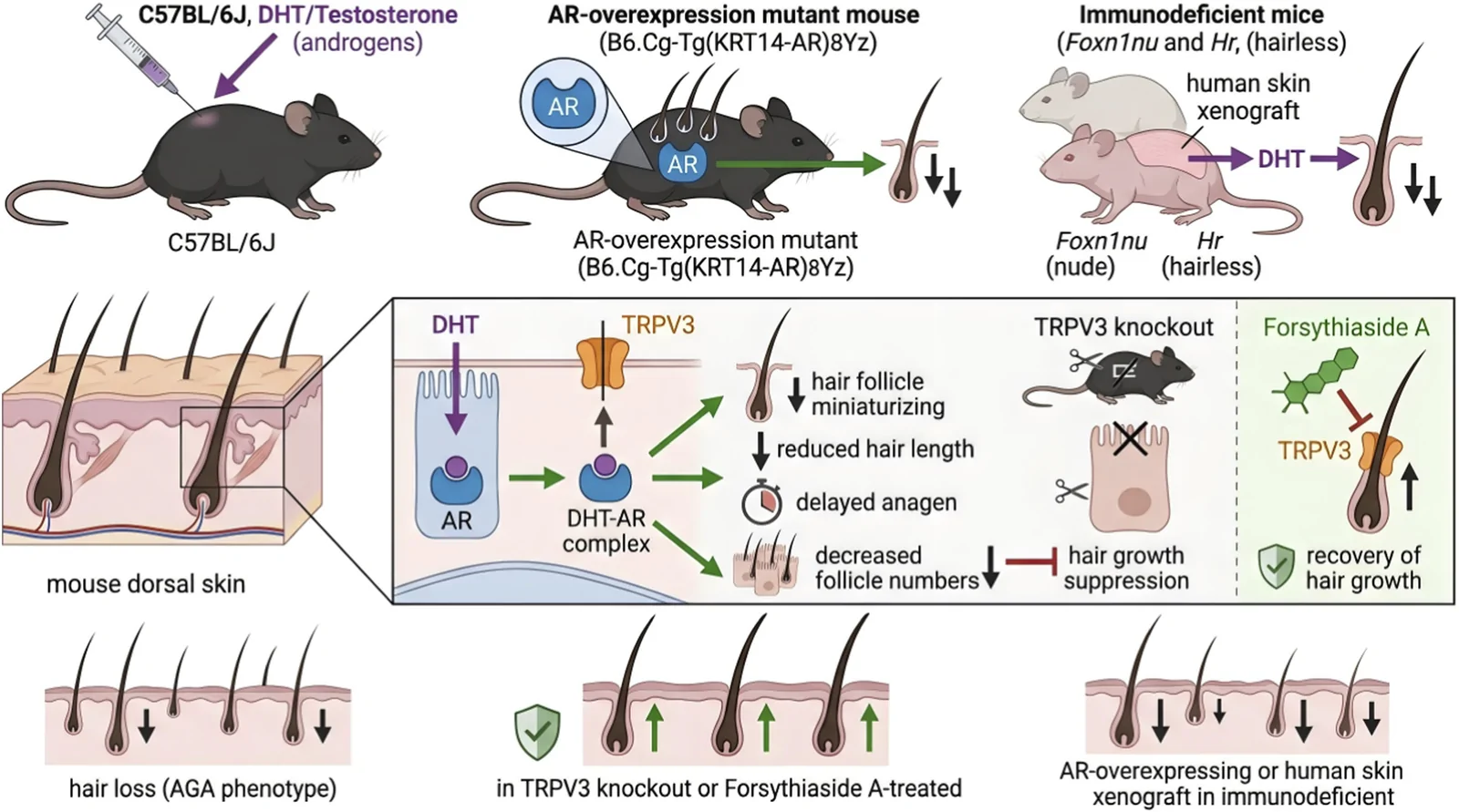
AGA mouse models, mechanistic validation, and TRPV3-targeted therapeutic strategies. This diagram illustrates three *in vivo* mouse models used to study the pathogenesis of AGA and explore potential therapeutic targets. It highlights that after DHT binds to the AR, activation of the TRPV3 ion channel drives hair follicle miniaturization and hair loss, and demonstrates that genetic knockout of TRPV3 or treatment with specific inhibitors can effectively reverse this process.

Mouse AGA models help explore AR signaling, a central pathway in AGA. DHT activates AR in dermal papilla cells, causing hair follicle regression, reversible by AR antagonists like *bicalutamide* in C57BL/6J mice. These models also study interactions with other pathways, such as Wnt/β-catenin, where DHT modulates β-catenin signaling in a concentration-dependent way: higher doses inhibit hair growth via downregulation of β-catenin and phosphorylated GSK-3β, while lower doses may promote growth, revealing a complex regulatory mechanism (Chen et al., 2020). The AGA mouse model is instrumental in evaluating new therapeutic agents, such as *Paeonia lactiflora* and *Poria cocos* extracts, which promote hair regrowth by lowering testosterone, reducing cytokines, and modulating hormone pathways (Zhang et al., 2021). Similarly, *Connatus semidecandrus* extract reduces androgen receptor expression and apoptosis, validating the model for testing anti-alopecia compounds (Jang et al., 2022). Advances in drug delivery systems have been tested in mouse models, such as minoxidil nanoparticles targeting hair follicles, which enhance hair growth more effectively than conventional formulations due to increased drug accumulation in the hair bulge and activation of hair follicle stem cells (Oaku et al., 2022). Likewise, microneedle patches with minoxidil-loaded microspheres provide sustained drug release and mechanical stimulation, leading to superior hair regeneration and reduced dosing frequency (Yin et al., 2023). Additionally, the mouse model has been used to study stress-related glucocorticoids’ effects on AR activity in dermal papilla cells, with findings showing that dexamethasone induces AR expression and nuclear translocation, potentially contributing to stress-induced hair loss (Kwack et al., 2022).

### 3.2 Other animal models of AGA

Although the development of treatments for androgenetic alopecia (AGA) primarily relies on mouse models, early research also explored non-human primate and non-mammalian models (Figure 4). Non-human primates, being phylogenetically close to humans, can effectively predict compound efficacy. AGA is well-defined in several primate species, characterized by progressive hair loss after puberty. Among these, the stump-tailed macaque is the most commonly used, as its hair loss closely resembles the human presentation and can be prevented by 5α-reductase inhibitors and androgen receptor blockers. Minoxidil promotes hair growth in macaques, but the effect subsides after discontinuation. The regulatory effect of androgens on skin appendages is not unique to mammals (Devja et al., 2023). Birds were once used to study the regulation of skin appendage cycling, but by contemporary standards they should be regarded as historical models, with their value lying primarily in evolutionary biology and comparative endocrinology. In the mid-20th century, researchers observed molting in certain avian species during the breeding season. For example, wattled starlings underwent molting after exogenous androgen supplementation and regenerated feathers upon withdrawal. Although domestic chickens do not exhibit androgen-dependent molting, they show pronounced feather sexual dimorphism, and the spontaneous “henny feathering” mutation can cause genetically male chickens to develop female-type plumage (George and Wilson, 1980). This phenotype was later confirmed to result from ectopic overexpression of aromatase in the skin, thereby advancing research on sex hormone-dependent cutaneous phenotypes and peripheral aromatase function (Wilson et al., 1987).

**FIGURE 4:**
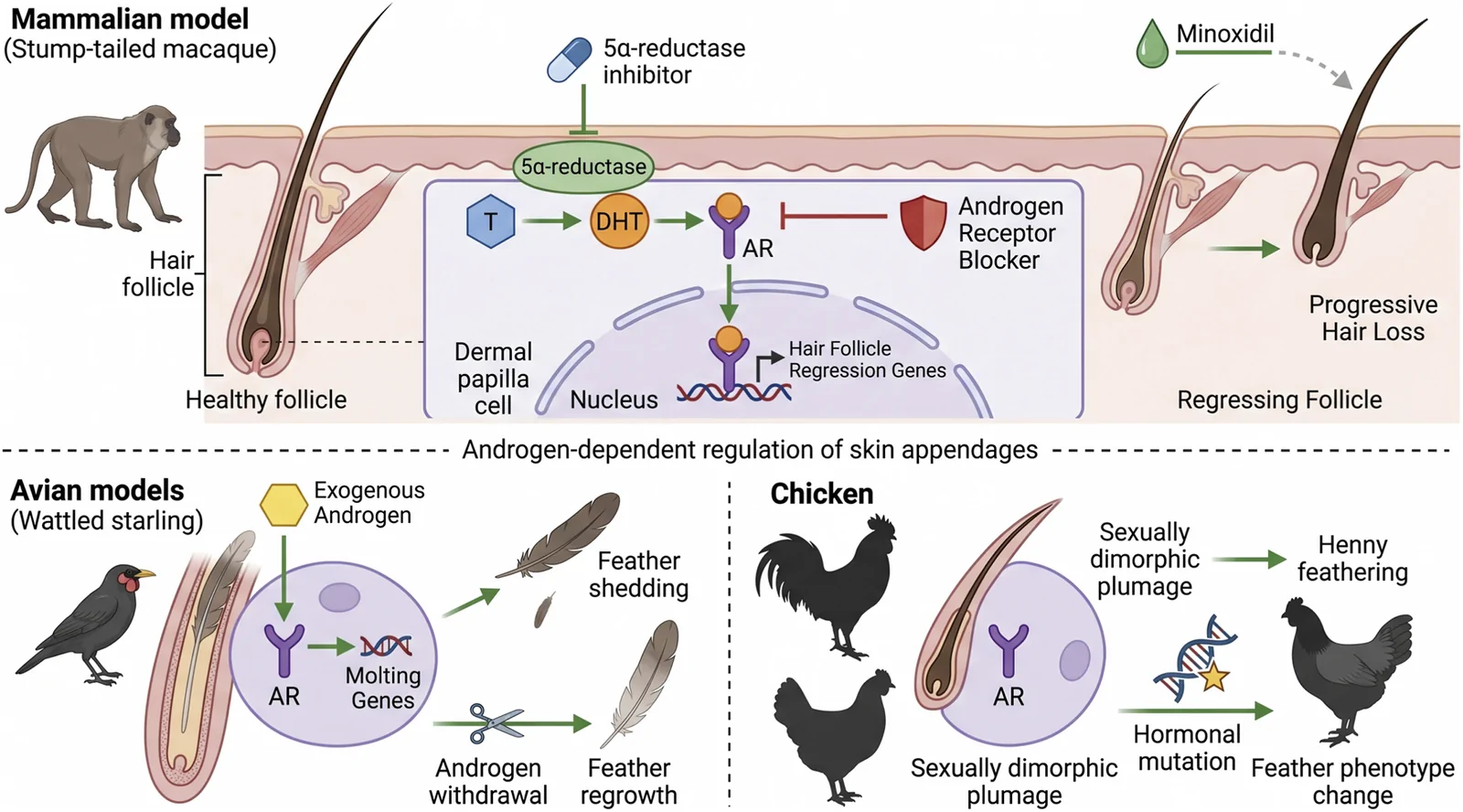
Other animal models of AGA, mechanisms, and intervention strategies. This diagram compares mammalian (stump-tailed macaque) and avian (myna, chicken) models to illustrate the shared molecular pathways through which androgens (primarily DHT) regulate hair and feather growth via intracellular AR signaling. It also highlights the action sites of targeted drugs (5α-reductase inhibitors, androgen receptor antagonists) and a hair regrowth agent (minoxidil) along this pathway.

### 3.3 Advantages and limitations of *in vivo* animal models

Currently, mouse models are the predominant tools in AGA research, widely utilized for drug screening and mechanistic investigations due to their well-defined genetic background, rapid reproduction, and relatively low cost (Fu et al., 2021). However, their inherent limitations must be recognized. First, there are marked interspecies differences in hair follicle biology (Stenn and Paus, 2021). Human follicles are large and extend deep into the dermis and subcutaneous fat, whereas mouse follicles are small, superficial, and comprise multiple subtypes. Moreover, the human anagen phase lasts 2–6 years and cycles asynchronously, while the mouse anagen phase lasts only about 3 weeks and exhibits synchronized “wave-like” cycling in early life, making it difficult to recapitulate the chronic follicular miniaturization characteristic of AGA. Mouse follicles also lack regional heterogeneity, limiting spatial analyses. In human follicles, both type 1 and type 2 5α-reductase isoenzymes are expressed, whereas mouse skin predominantly expresses type 1, with very low type 2 expression; consequently, drugs targeting type 2 5α-reductase are unlikely to achieve adequate inhibitory efficacy in mice. In addition, mouse Akr1c21 has no direct ortholog in the human AKR1C family, and their substrate preferences and catalytic directions differ. Second, fundamental differences exist in phenotype induction and natural disease course (Trüeb, 2002). Human AGA progresses slowly in an age- and site-dependent manner, whereas most mouse models rely on acute exposure to exogenous androgens (4–12 weeks) to induce hair loss, which fails to reproduce the chronic disease progression. Dosing protocols are highly heterogeneous, with testosterone doses varying by nearly 10-fold, along with diverse administration routes and durations. High-dose androgens may also confound evaluation through systemic effects. Collectively, mouse models remain valuable for mechanistic exploration, but extrapolation to human AGA requires caution; findings should be corroborated using humanized models, three-dimensional hair follicle organ culture, and clinical samples.

Non-human primates, such as the stump-tailed macaque, are regarded as ideal homologous models for AGA because of their high physiological and genetic homology with humans. Early studies confirmed that they can be used to accurately evaluate the hair growth-promoting effects of drugs, such as prostaglandin analogs. Nevertheless, this model has significant limitations: difficulty in acquisition, high housing and maintenance costs, strict ethical constraints, and a lack of updated research in over 2 decades, all of which restrict its current application.

Avian molting is fundamentally distinct from human AGA. Molting is a physiological, cyclic, and reversible process of feather replacement regulated by seasonal and hormonal cues, whereas AGA is a pathological, irreversible process of follicular miniaturization accompanied by chronic inflammation and fibrosis (Paus and Cotsarelis, 1999). The two differ fundamentally in structure, mechanism, and clinical significance, and equating molting with hair loss is conceptually flawed. Avian models also face practical limitations, including poor species accessibility, complex husbandry requirements, and a lack of molecular tools, leaving research largely confined to phenotypic description (Lee et al., 2024; Hosny et al., 2026). Therefore, avian models should no longer be used to study AGA pathogenesis or evaluate therapeutic agents, and their relevance is limited to comparative studies of evolutionary and developmental biology (Dhouailly, 2009).

## 4 *Ex vivo* AGA model

### 4.1 *Ex vivo* hair follicle organ culture model

Another pivotal tool in AGA research is the *ex vivo* human hair follicle organ culture model. This system maintains intact hair follicles acquired from scalp biopsies of healthy individuals or AGA patients, preserving their native architecture and physiological functions. By providing a controlled environment, it allows researchers to simulate hair follicle cycling phases *in vitro*—a function that is critical for isolating the direct effects of pharmacological agents on hair growth dynamics, free from systemic confounders. This model must be distinguished into two culture systems (Figure 5): conventional microdissection-based isolated hair follicle culture and full-thickness scalp organ culture. The former retains core structures such as the dermal papilla and dermal sheath, but often fails to fully preserve the dermal environment and adnexal structures such as sebaceous glands (Langan et al., 2015). The latter retains full-thickness scalp architecture, better mimicking the androgen-responsive microenvironment, yet suffers from short culture survival and limited nutrient diffusion (Stenn and Paus, 2021). Sebaceous glands are highly androgen-responsive organs that express 5α-reductase and AKR family members and can locally regulate DHT concentrations. Studies have shown that sebaceous gland cells are key regulators of cutaneous androgen homeostasis (Fritsch et al., 2001). Therefore, the absence of sebaceous glands in isolated hair follicle cultures may lead to underestimation of their influence on drug efficacy. As an example, the key androgen DHT, which is implicated in AGA pathogenesis, suppresses hair growth in these cultured follicles. Importantly, this suppression can be reversed by agents such as 1′S-1′-Acetoxychavicol Acetate (ACA). These findings underscore the model’s value in evaluating the potential of anti-AGA therapies (Park et al., 2025). In addition to drug testing, this organ culture system is suitable for evaluating the delivery efficiency and short-term transgene expression of locally administered agents, including exosomes, growth factors, and gene therapy vectors (Hoffman, 2000). However, it has inherent limitations in assessing the long-term efficacy, safety, and effects across multiple hair follicle cycles of gene therapy, because organ-cultured hair follicles can be maintained for only a limited period and do not support full hair cycle progression (Langan et al., 2015). Therefore, *ex vivo* results should be interpreted in conjunction with *in vivo* model data to validate durable therapeutic outcomes (Fu et al., 2021; Lee et al., 2024).

**FIGURE 5:**
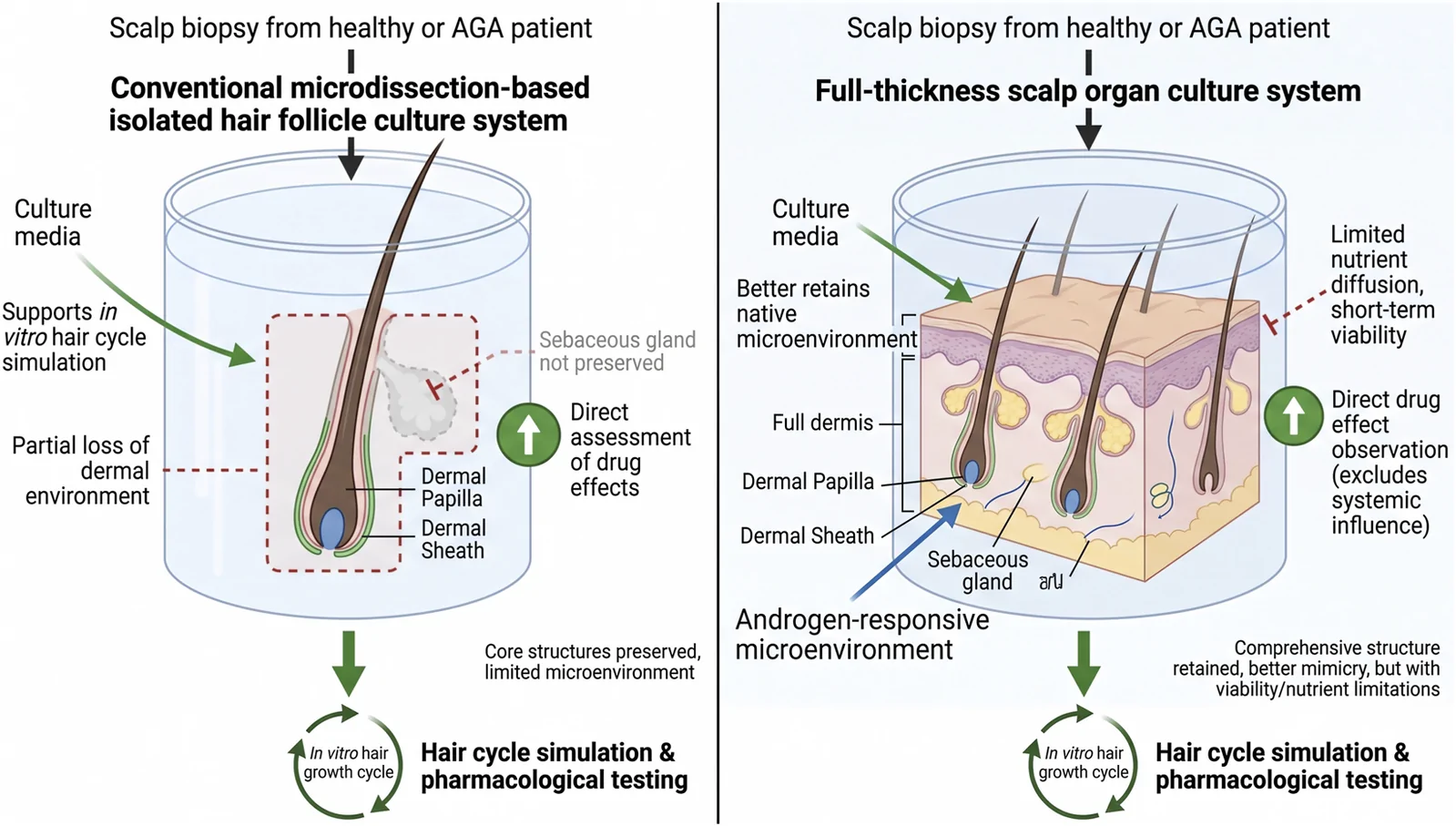
*Ex vivo* hair follicle organ culture models of AGA and intervention strategies. This diagram illustrates an *ex vivo* hair follicle organ culture model derived from scalp biopsy specimens, and outlines the experimental framework covering DHT-induced hair loss mechanisms, drug blockade effects, and novel regenerative therapies (exosomes, growth factors, and gene therapy).

### 4.2 Advantages and limitations of hair follicle organ culture model

The *ex vivo* hair follicle organ culture model is an experimental system in which intact hair follicle units are isolated from the skin and maintained in specific culture media *in vitro*. It occupies a unique position in hair research, such as AGA, offering both significant advantages and inherent limitations.

The main advantages include preserving the complete three-dimensional structure of the hair follicle, key cellular components (e.g., dermal papilla cells, hair matrix keratinocytes, and outer root sheath keratinocytes, etc.), and the extracellular matrix, thereby enabling the simulation of complex *in vivo* cell–cell interactions and signaling. This model facilitates direct observation and intervention in cycle dynamics by enabling the monitoring of hair growth and morphological changes within a limited culture period, although it does not entirely replicate the physiological hair cycle (Stenn and Paus, 2021). Furthermore, since it is derived directly from human tissue, experimental results typically have higher predictive value for humans than animal models, making it suitable for mechanistic studies and drug screening (Laufer-Britva et al., 2025).

Key disadvantages include technical challenges and limited accessibility: hair follicle isolation requires meticulous skill, and human scalp samples are difficult to obtain. The culture survival time is limited, usually ranging from days to weeks, making it hard to support long-term studies. Moreover, the model is removed from *in vivo* systemic regulation (e.g., neural and endocrine signals), preventing the study of these systemic factors. Besides, sebaceous glands are highly androgen-responsive organs that express 5α-reductase and AKR family members and can locally regulate DHT concentrations. Studies have shown that sebaceous gland cells are key regulators of cutaneous androgen homeostasis (Fritsch et al., 2001). Therefore, the absence of sebaceous glands in isolated hair follicle cultures may lead to underestimation of their influence on drug efficacy. Natural variability among different hair follicles affects experimental uniformity, and ultra-high-throughput drug screening is not feasible (Platt et al., 2022).

In summary, this model serves as a critical bridge between monolayer cell culture and whole-animal experiments, particularly suitable for studying hair follicle pathophysiology and validating local drug effects at a tissue level close to humans. However, its limitations dictate that it typically needs to be combined with other models to form a complete chain of research evidence.

## 5 Application of AGA models in drug screening and mechanism research

### 5.1 Natural compound screening

The use of natural compounds for treating AGA has attracted considerable interest. Researchers are leveraging established AGA models to validate the anti-hair loss potential of these compounds, which are thought to act through diverse molecular mechanisms.

Fucoidan, a sulfated polysaccharide extracted from brown seaweed, exemplifies this category of natural compounds. It has demonstrated efficacy in reversing testosterone propionate-induced hair follicle atrophy and pigment loss. This effect is mediated primarily through two key mechanisms: modulation of the Wnt/β-catenin signaling pathway and enhancement of vascular endothelial growth factor release, which promotes angiogenesis and follicular nourishment. The restoration of both follicular structure and pigmentation underscores fucoidan’s therapeutic promise in AGA models, highlighting its dual role in stimulating follicular proliferation and improving the follicular microenvironment (Wang Z. et al., 2025).

Similarly, broccoli sprout extract (BSE)—rich in glucosinolates like thio-glucosides—has demonstrated remarkable hair restoration efficacy in AGA mouse models, achieving up to 99% hair recovery. This effect is attributed to the upregulation of DHT-metabolizing enzymes, including Akr1c21 and Dhrs9, in both liver and skin tissues. These enzymatic changes facilitate enhanced androgen metabolism and thereby reduce follicular androgenic insult. Furthermore, BSE concurrently activates the Wnt/β-catenin pathway, which further supports follicular regeneration. This indicates a synergistic mechanism that involves both androgen metabolism modulation and stem cell signaling activation (Subedi et al., 2025).

Another promising natural formulation, Changfa Zirong Powder, a traditional Chinese medicine formula, activates the Wnt/β-catenin pathway and upregulates the expression of β-catenin and protein phosphatase 2A. It significantly improves symptoms in the AGA mouse model by promoting hair regrowth, skin darkening, hair shaft elongation, dermal thickness, and local hormonal balance without affecting systemic levels (Chen et al., 2026).

Collectively, these findings highlight the multifaceted mechanisms through which natural compounds counteract AGA pathogenesis. By targeting androgen metabolism, key regenerative signaling pathways (e.g., Wnt/β-catenin), and metabolic regulators, these agents work to restore follicular function. Integrating such natural compounds into therapeutic regimens presents a promising strategy for safer, multi-targeted interventions in AGA management. However, further clinical validation and mechanistic elucidation are warranted to fully realize their potential.

### 5.2 Signaling pathway research

AGA models have served as essential tools for dissecting the complex signaling pathways that govern hair follicle miniaturization and regeneration. The insights derived from these models are critical for the development of targeted therapies. Notably, the pathways discussed below were each identified or validated through a combination of complementary model systems—typically DHT-treated dermal papilla cells (DPCs) for early molecular mechanistic dissection, *ex vivo* human hair follicle organ culture for confirmation at the intact follicle level, and *in vivo* animal models for overall phenotypic validation. This model hierarchy has been instrumental in translating molecular observations into pathophysiological understanding.

The Wnt/β-catenin pathway is recognized as a central axis in hair follicle regeneration. Its activation promotes DPCs proliferation and the differentiation of hair follicle stem cells, thereby contributing to anagen phase maintenance and follicular cycling. The initial evidence linking androgen signaling to Wnt inhibition was obtained in DHT-treated DPCs, where androgen exposure suppresses nuclear β-catenin accumulation and downregulates Wnt-responsive gene transcription (Leirós et al., 2012). These molecular findings were subsequently corroborated in *ex vivo* human hair follicle organ culture, where β-catenin pathway activation partially counteracts DHT-induced follicular regression, and ultimately confirmed in *in vivo* AGA mouse models, where experimental activation of the pathway enhances follicular stem cell activity and counteracts androgen-induced follicular regression (Shang et al., 2024). This multi-model progression illustrates how cell-based assays define molecular mechanisms, while *ex vivo* and *in vivo* systems establish their functional relevance.

Conversely, the transforming growth factor-beta 1 (TGF-β1)/SMAD2 signaling cascade plays a pivotal role in driving DHT-induced senescence of DPCs, a process that contributes to follicular miniaturization. The identification of this pathway relied primarily on DHT-treated DPCs (*in vitro*), in which TGF-β1 secretion and SMAD2 phosphorylation are markedly upregulated, leading to cellular senescence, growth arrest, and impaired proliferative capacity (Inui et al., 2002). Notably, this damage can be countered through exosome-mediated delivery of microRNAs that target the TGF-β1/SMAD2 pathway; this intervention not only reverses DPCs damage *in vitro* but also improves hair growth in *in vivo* AGA mouse models, highlighting a novel intercellular communication mechanism capable of mitigating androgenic follicular aging (Ma et al., 2025). Thus, *in vitro* assays pinpointed the molecular target, whereas the *in vivo* model was essential for demonstrating the therapeutic potential of pathway modulation.

Autophagy, a cellular homeostatic process, is increasingly recognized for its involvement in AGA pathogenesis. The functional significance of autophagy in AGA was chiefly established in *in vivo* AGA mouse models, where PRP treatment enhances autophagic activity, as evidenced by the upregulated expression of autophagy markers LC3 and Beclin-1, which correlates with improved hair growth. The causal role of autophagy in follicular regeneration was further confirmed by the observation that PRP efficacy is attenuated by autophagy inhibitors such as 3-methyladenine (Xiao et al., 2025). Complementary *in vitro* experiments in cultured DPCs and keratinocytes have additionally linked autophagic flux to cell proliferation and survival under androgenic stress, providing mechanistic support for the *in vivo* findings. This pathway exemplifies the necessity of *in vivo* models for validating phenotype-level efficacy, while cellular assays clarify the underlying molecular causality.

Beyond those previously discussed, several other signaling pathways contribute to AGA molecular pathology. The RAS/ERK pathway influences cell proliferation and differentiation within the follicular microenvironment, with evidence derived largely from *in vitro* DPCs proliferation assays and *ex vivo* follicle cultures. The mTOR signaling integrates nutrient and growth factor cues to regulate follicle cycling, as demonstrated by pharmacological modulation in both cultured follicular cells and organ-cultured human hair follicles. Additionally, the transient receptor potential vanilloid 3 (TRPV3) channels—expressed in keratinocytes—modulate calcium influx and may thereby affect follicular keratinization and homeostasis; this finding originates from keratinocyte cell culture studies and awaits further validation in *ex vivo* and *in vivo* AGA models. These examples further underscore the complementary strengths of *in vitro*, *ex vivo*, and *in vivo* systems in building a comprehensive picture of AGA signaling.

To conclude, these pathways form a complex regulatory network that orchestrates hair follicle biology; their dysregulation is a fundamental driver of AGA progression. We summarize the experimental models and methodological contributions used to investigate these signaling pathways in AGA (Table 2). Studying these mechanisms through AGA models provides crucial insights: it not only elucidates the pathophysiology of the disease but also unveils novel molecular targets. This understanding is essential for developing precision therapies aimed at restoring hair growth and follicular integrity.

**TABLE 2:** Signaling pathways in AGA: experimental model systems and their methodological contributions.

Pathway	*In vitro* (DPCs/keratinocytes)	*Ex vivo* (hair follicle organ culture)	*In vivo* (AGA animal models)	Model contribution
Wnt/β-catenin	DHT suppresses nuclear β-catenin accumulation and downregulates Wnt-responsive gene transcription (Leirós et al., 2012)	Activation of the pathway partially counteracts DHT-induced follicular regression	Experimental activation enhances follicular stem cell activity and blocks androgen-induced regression (Shang et al., 2024)	Cell-based assays identified the molecular mechanism; *ex vivo* and *in vivo* systems established functional relevance
TGF-β1/SMAD2	DHT upregulates TGF-β1 secretion and SMAD2 phosphorylation, leading to DPC senescence (Inui et al., 2002)	—	Exosome-delivered microRNAs targeting this pathway reverse DPC damage and improve hair growth (Ma et al., 2025)	*In vitro* pinpointed the molecular target; *in vivo* demonstrated therapeutic potential
Autophagy	Autophagic flux supports DPC/keratinocyte proliferation and survival under androgenic stress	—	PRP upregulates LC3/Beclin-1 and improves hair growth; autophagy inhibitors (e.g., 3-MA) attenuate this effect (Laufer-Britva et al., 2025)	*In vivo* established phenotype-level efficacy; *in vitro* clarified mechanistic causality
Other pathways (RAS/ERK, mTOR, TRPV3)	RAS/ERK: DPC proliferation assays; mTOR: follicular cell pharmacology; TRPV3: keratinocyte calcium influx studies	RAS/ERK evidence obtained in *ex vivo* follicle cultures; mTOR modulation confirmed in organ-cultured human hair follicles	Further validation awaited, particularly for TRPV3	Illustrates how complementary *in vitro*, *ex vivo*, and *in vivo* models together construct a comprehensive signaling network

### 5.3 Stepwise validation pipeline and decision tree

No single AGA model fully recapitulates human disease. We propose a stepwise validation pipeline (Figure 6) with three platforms: *in vitro* cell models, *ex vivo* hair follicle culture, and *in vivo* models. Model-specific limitations must be acknowledged at each step.

Step 1: Human *in vitro* cell models. DHT-treated DPCs, follicular keratinocytes, and related cells offer a rapid, reproducible platform for mechanistic studies and compound screening. Positive results indicate direct cell-autonomous effects, such as Wnt/β-catenin or TGF-β/SMAD2 pathway modulation. However, lacking 3D follicular architecture, positive findings are preliminary, and negative results may not rule out *in vivo* activity due to metabolic activation or niche dependence. Avoid premature termination based solely on *in vitro* data.
Step 2: *Ex vivo* human hair follicle organ culture. Microdissected free hair follicles or full-thickness scalp organ culture retain key structures like the dermal papilla, hair matrix, root sheaths, and sebaceous glands. These systems enable tissue-level analysis of hair shaft growth and signaling. Positive results offer proof-of-concept for early hair-cycle effects only, given limited culture duration. Negative outcomes may stem from technical limits, and deprioritization is reasonable, though *in vivo* confirmation may still be needed for systemic or immune-mediated candidates.
Step 3: *In vivo* animal models. After passing *ex vivo* testing, *in vivo* models like androgen-sensitive mice, human scalp xenografts, or genetically modified strains assess whole-organism effects on hair cycling, follicular miniaturization, androgen metabolism, and safety. Positive results are strong preclinical evidence, but species differences require caution: rodent hair cycling and androgen dependence differ from human biology, and xenografts often lack an intact immune system. Positive findings should be cross-validated with human *ex vivo* data for translational relevance, while negative outcomes should be interpreted cautiously due to model-specific limitations, and complementary models or delivery strategies should be explored before final dismissal.

**FIGURE 6:**
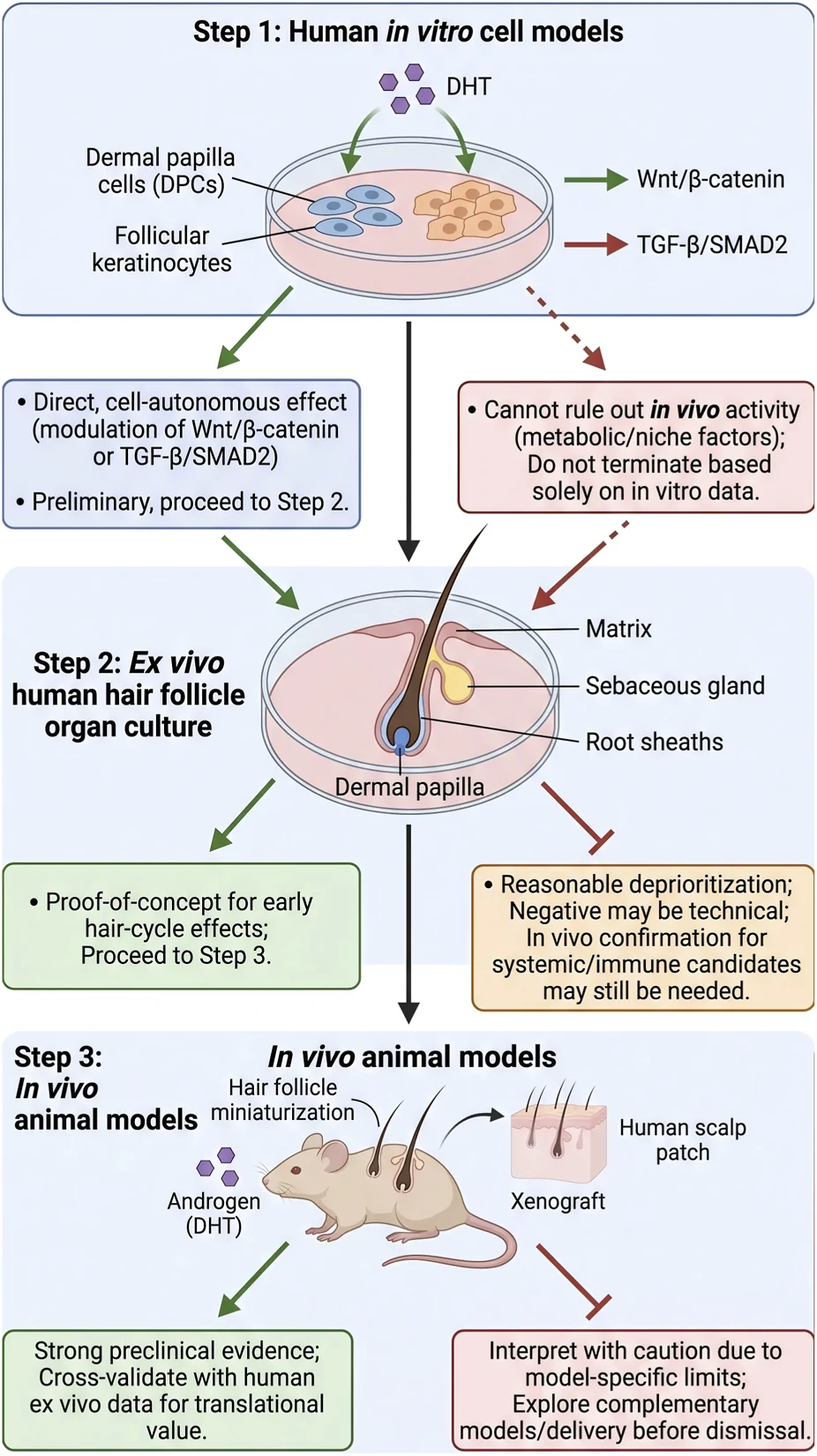
Stepwise validation pipeline with three platforms of AGA models. Step 1 uses human dermal papilla and keratinocyte cultures to test direct DHT-driven Wnt/β-catenin and TGF-β/SMAD2 effects; positive results move to Step 2, while negatives do not rule out *in vivo* activity. Step 2 employs *ex vivo* human hair follicle organ culture to assess early hair-cycle changes (matrix, sebaceous, sheaths, papilla); proof-of-concept advances to Step 3, but negative outcomes may be technical and require *in vivo* testing for systemic/immune candidates. Step 3 uses human scalp xenografts with DHT to generate strong preclinical evidence; cross-validate with *ex vivo* data and explore complementary models/delivery routes before final dismissal.

This stepwise pipeline emphasizes the complementary strengths and inherent limitations of each model level (Table 3). By requiring explicit acknowledgment of model constraints, the decision tree helps researchers minimize false-positive and false-negative conclusions, thereby strengthening the translational relevance of AGA model research.

**TABLE 3:** Model-specific limitations and their impact on data interpretation in the stepwise validation pipeline.

Model level	Key advantages	Key limitations	Impact on data interpretation
Human *in vitro* cell models	High-throughput, well-controlled, human-derived, suitable for mechanistic dissection	Lack of three-dimensional follicular architecture; no hair cycle progression; may underestimate metabolic transformation	Positive results indicate only direct cell-autonomous effects; negative results do not exclude *in vivo* activity and require cautious decision-making
*Ex vivo* human hair follicle organ culture	Preserves follicular architecture; allows tissue-level functional assessment	Limited culture duration; cannot support full hair cycling; sebaceous glands may be absent	Positive results reflect only early hair-cycle events; negative results may be confounded by technical factors and should prompt optimization and retesting
*In vivo* animal models	Allows evaluation of hair cycling, follicular miniaturization, systemic androgen metabolism, and safety	Species-specific differences; incomplete immune environment; model induction differs from human disease	Positive results should be cross-validated with human *ex vivo* data; negative results may be model-specific and require exclusion before a final conclusion

## 6 Clinical assessment of AGA model

### 6.1 Similarities between models and human AGA

An ideal AGA model should faithfully recapitulate the key pathological hallmarks observed in human disease, including follicular miniaturization, shortened anagen phase, androgen dependency, and the inflammatory microenvironment. Since no single model can perfectly encapsulate the full spectrum of human AGA, each model has its own advantages and limitations, necessitating a systematic evaluation based on the key dimension of similarity to human pathology (Table 1).

*In vitro* cell models are highly simplified systems and thus require validation in more complex settings. Nevertheless, they remain fundamental for dissecting androgen receptor (AR) signaling and for initial drug target screening, making them essential tools for uncovering molecular mechanisms at the cellular level.

Among animal models, mice have become the primary choice for *in vivo* mechanistic research, largely because they effectively replicate the core androgen-dependent processes and are relatively cost-effective. Non-human primates show the closest similarity to humans in terms of pathological phenotype and treatment response, yet practical constraints have prevented their widespread adoption. Avian models, on the other hand, mainly support broad principles of hormonal regulation and offer limited insight into the specific pathology of AGA. By contrast, the *ex vivo* hair follicle organ culture model—which relies directly on intact human tissue—acts as a crucial bridge between cellular and animal studies. It most closely mirrors how drugs or factors affect human hair follicles in their native structure, giving it particularly high clinical predictive value.

In practice, these models actually work together—they form a kind of synergistic, stepwise, and complementary system. It often starts with *in vitro* cell models, which are used for high-throughput screening and pathway analysis to help uncover mechanisms. Then, researchers might move to *ex vivo* human hair follicle organ culture models to check how those findings play out in intact tissue. Finally, animal models like mice come into the picture, providing a way to gauge systemic efficacy, pharmacokinetics, and potential safety issues. Therefore, choosing the right model really should be driven by what you’re trying to do—whether it’s nailing down a molecular mechanism, running initial drug screens, or confirming efficacy. In many cases, the best approach is not to rely on just one model, but to combine several, building a more complete evidence chain from target discovery right through to preclinical research.

### 6.2 Translational medical value of models

The translational utility of AGA models in drug development hinges on their predictive validity for clinical efficacy and safety.

Established pharmacotherapies, such as minoxidil and finasteride, have shown consistent therapeutic effects across both animal models and human clinical trials. This underscores the capacity of these models to recapitulate key disease pathways and predict treatment outcomes. For instance, topical finasteride formulations delivered via biomimetic nanovesicles have demonstrated enhanced skin permeation and hair regrowth in testosterone-induced AGA mouse models, paralleling clinical benefits observed in patients (Wu et al., 2024). Similarly, novel topical androgen receptor antagonists identified through murine studies have exhibited potent hair growth promotion without systemic toxicity, highlighting the models’ role in optimizing drug candidates (Zhang et al., 2024).

However, some compounds that are efficacious in animal models fail to translate into clinical success. These challenges advocate for an integrated multi-model strategy that combines *in vitro* human follicle cultures, *ex vivo* organ cultures, and *in vivo* animal models to comprehensively evaluate pharmacodynamics, pharmacokinetics, and safety profiles. Promising advances—such as patient-derived iPSC-based hair follicle organoids and humanized mouse models—offer ways to enhance clinical predictability by incorporating patient-specific genetic and microenvironmental factors (McElwee and Sundberg, 2025). Furthermore, innovative delivery systems like microneedle patches and hydrogel formulations have been developed and validated in preclinical models, showing encouraging translational potential (Xing et al., 2025).

In summary, while current models provide valuable platforms for AGA drug discovery, their translational value can be significantly augmented through multi-model integration and the incorporation of patient-derived systems, ultimately accelerating the development of effective and safe therapeutics.

## 7 Future development directions and challenges of models

### 7.1 Three-dimensional organoids and organ-on-a-chip technologies

Three-dimensional (3D) hair follicle organoid models represent a pivotal frontier in AGA research, offering the potential to recapitulate the complex cyclical regeneration and microenvironmental dynamics of hair follicles *in vitro*. These models are constructed by co-culturing hair follicle stem cells (HFSCs), DPCs, and surrounding matrix cells within microfluidic chips or bioprinted scaffolds, thereby mimicking the essential epithelial-mesenchymal interactions required for hair follicle cycling (Ma et al., 2023). This ability to simulate periodic regeneration is crucial, as androgenic disruption in AGA primarily impairs HFSCs differentiation via deregulated DPC-secreted factors (Ceruti et al., 2021). Furthermore, organ-on-a-chip technology integrates multiple cell types, vascular networks, and mechanical stimuli to achieve greater physiological fidelity than traditional 2D cultures. Although these platforms have been preliminarily applied in skin disease studies, their adaptation to AGA remains nascent, lacking full maturity and standardization. Nonetheless, standardized 3D organoid models are anticipated to reduce reliance on animal testing and enhance drug screening efficiency by providing more physiologically relevant platforms. They enable direct investigation of AR signaling, DHT effects, and paracrine factors (e.g., BMP2, DKK-1) implicated in miniaturization and apoptosis (Ceruti et al., 2021; Kwack et al., 2020). Additionally, 3D cultures of matched frontal and occipital DPC lines have revealed differential gene expression and androgen responsiveness, underscoring the importance of spatially relevant models (Kwack et al., 2023). The integration of microfluidics and bioprinting promises to recapitulate the follicular niche, enabling dynamic studies of hair cycle phases and therapeutic interventions. However, challenges persist in achieving full follicular morphogenesis, vascularization, and immune cell incorporation. Overall, 3D organoid and organ-on-a-chip technologies are poised to revolutionize AGA research by providing scalable, physiologically relevant models that bridge the gap between *in vitro* studies and clinical applications.

It is important to distinguish proof-of-concept models from clinically predictive models when interpreting results obtained from current 3D organoid and organ-on-a-chip platforms for AGA. Although these platforms recapitulate selected epithelial–mesenchymal interactions among dermal papilla cells, keratinocytes, vascular cells, and immune cells (Lee et al., 2020), they have not yet fully reconstructed the intact architecture of the human hair follicle, nor have they faithfully reproduced the androgen-driven follicular miniaturization that constitutes the core pathological feature of AGA (Abaci et al., 2018). Consequently, claims regarding the translational maturity of these technologies should remain cautious; at present, they are best viewed as complementary proof-of-concept tools that require further development and validation before they can serve as predictive models for clinical response (Huh et al., 2011).

In light of these limitations, future 3D organoid or organ-on-chip AGA models may also benefit from the incorporation of sebocytes or sebaceous gland components, in addition to the currently emphasized dermal papilla cells, keratinocytes, vascular cells, and immune cells (Hoffman, 2000). The sebaceous gland is an integral part of the pilosebaceous unit and actively participates in local androgen metabolism. Human sebocytes express a broad repertoire of steroidogenic enzymes, including type 1 5α-reductase and multiple 17β-hydroxysteroid dehydrogenases, and are regarded as key regulators of androgen homeostasis in human skin (Courchay et al., 1996). Of particular translational relevance, members of the aldo-keto reductase 1C (AKR1C) family—especially AKR1C2—catalyze the inactivation of dihydrotestosterone (DHT) to 5α-androstane-3α, 17β-diol in cutaneous tissues (Penning et al., 2000). Accumulating evidence suggests that the expression of these DHT-inactivating AKR1C enzymes in human scalp sebaceous glands declines with advancing age (Ikuno et al., 2026). This age-related loss of local androgenic buffering capacity may contribute to the age-dependent onset and progression of AGA. Models that omit the sebaceous compartment are therefore unlikely to capture these age-related changes in the local androgen microenvironment. We propose that next-generation AGA 3D organoid or organ-on-chip systems should integrate sebocytes—for example, through co-culture with induced pluripotent stem cell (iPSC)-derived sebocyte-like cells or incorporation of microdissected sebaceous glands—to recreate the local steroidogenic niche and to investigate how sebaceous gland aging affects androgen metabolism and follicular miniaturization (Zouboulis et al., 2008).

### 7.2 Multi-omics integration and precision modeling

The emergence of multi-omics technologies—such as single-cell RNA sequencing, spatial transcriptomics, and proteomics—has revolutionized the understanding of AGA. These tools enable high-resolution mapping of cellular heterogeneity and molecular dysregulation within hair follicles. For instance, single-cell transcriptomic studies of patient follicles have identified key cell subpopulations and dysregulated signaling pathways—including Wnt/β-catenin, BMP, and androgen receptor signaling—that govern stem cell fate and cycling (Cuevas-Diaz Duran et al., 2024). Spatial transcriptomics further clarifies immune microenvironment alterations, revealing a peri-infundibular infiltration of Th2-biased CD4^+^ T cells that may promote follicular miniaturization and inflammation (Matsusaka et al., 2022). Integrated multi-omics approaches, combining analysis of interaction networks and pathway enrichment, have directly pinpointed dysregulated lipid metabolism pathways and key candidate genes like ACAA1 and IDH1, suggesting novel therapeutic targets (Yang et al., 2026). The integration of these diverse datasets paves the way for constructing precise, patient-specific AGA models that capture the complex interplay of genetic, epigenetic, and environmental factors.

Gene editing tools, particularly CRISPR/Cas9, enable the creation of cellular and animal models carrying specific mutations in key genes such as FGFR3 and AR, thereby allowing functional dissection of genetic contributions to AGA pathogenesis. In parallel, artificial intelligence (AI)-assisted modeling can optimize experimental parameters and predict therapeutic responses by analyzing large-scale multi-omics data, enhancing both model fidelity and translational relevance. The integration of multi-omics data with gene editing and AI thus establishes a powerful framework for developing precision AGA models that capture patient heterogeneity and support personalized therapeutic screening.

It is also important to recognize the technical limitations of multi-omics approaches when interpreting transcriptomic data from AGA models. A representative challenge is the accurate quantification of highly homologous gene families, such as the aldo-keto reductase 1C (AKR1C) family. The AKR1C1–AKR1C4 isoforms share high sequence identity, and short-read RNA sequencing (RNA-seq) often cannot unambiguously assign short reads to individual paralogous genes, leading to cross-mapping, multi-mapping, and inaccurate isoform-specific quantitation (Conesa et al., 2016). This limitation is particularly relevant to AGA research because AKR1C isoforms have distinct functions; for instance, AKR1C2 catalyzes the inactivation of DHT in cutaneous tissues, including the scalp sebaceous gland (Zouboulis et al., 2008). We therefore recommend that expression changes of AKR1C family members identified by RNA-seq be validated using targeted qPCR with isoform-specific primers or probes, as well as protein-level analyses using isoform-selective antibodies or targeted mass spectrometry (Bustin et al., 2025). Furthermore, emerging long-read RNA sequencing technologies, which provide full-length transcript information, can help resolve homologous gene families with greater accuracy and should be considered as complementary approaches in future AGA multi-omics studies (Amarasinghe et al., 2020).

### 7.3 Standardization and ethical challenges

Despite these advances, significant challenges persist in the standardization of AGA models. Variability in cell sources, culture conditions, induction protocols, and evaluation metrics hampers reproducibility and comparability across studies. For instance, differences in dermal papilla cell isolation, immortalization methods, and androgen responsiveness can lead to inconsistent results, as observed in matched frontal and occipital DPC lines (Matsusaka et al., 2022). To address these issues, international academic societies and industry consortia should establish comprehensive guidelines delineating best practices for model development, including standardized cell characterization, culture media composition, androgen induction protocols, and outcome measures such as hair follicle miniaturization and molecular marker expression.

The utilization of animal models in AGA research is increasingly scrutinized under ethical frameworks emphasizing the 3R principles—replacement, reduction, and refinement—to minimize animal use and suffering. This ethical imperative drives the development and adoption of alternative models, such as organoids and organ-on-a-chip systems, which offer human-relevant platforms without animal involvement. Ethical considerations also extend to clinical research, where patient consent, data privacy, and equitable access to emerging therapies must be ensured.

## 8 Conclusion

AGA model research has advanced significantly, deepening understanding of its pathophysiology and therapeutic strategies. The integration of *in vitro*, *in vivo*, and *ex vivo* systems has been instrumental in dissecting the complex interplay of hormonal, genetic, and environmental factors. Each model offers unique strengths and limitations: *in vitro* models enable high-throughput screening but lack native microenvironments; animal models provide systemic exposure but face species-specific translational barriers; *ex vivo* human follicle cultures maintain tissue architecture yet are constrained by limited viability. Emerging technologies, including exosome-based platforms, nanocarriers, and 3D organoids, enable patient-specific modeling and targeted delivery, though they require rigorous validation. The future lies in converging advanced bioengineering, multi-omics profiling, and AI to generate comprehensive disease signatures and predict treatment responses. Standardization and ethical compliance are critical for reproducibility and regulatory acceptance. Ultimately, balancing existing model strengths with emerging innovations, fostered by interdisciplinary collaboration and methodological harmon-ization, will yield more predictive, personalized, and effective interventions for AGA, setting a paradigm for precision dermatology.
